# A Practical Treatise on the Diseases of the Eye

**Published:** 1840-04

**Authors:** 


Art. XIII.—*A Practical Treatise on the Diseases of the Eye.* By William Mackenzie, m.d., Surgeon-Oculist in Scotland in Ordinary to her Majesty, Lecturer on the Eye in the University of Glasgow. *To which is prefixed an Anatomical Introduction, Explanatory of a Horizontal Section of the Human Eyeball.* By Thomas Wharton Jones, Surgeon. Third edition, with two copper-plates, and numerous woodcuts.—*London*, 1840. 8vo, pp. 923.

We have had for some time in preparation a general article on ophthalmology, but the repeated accession of new books to our list has hitherto interfered with its completion. The work before us is, however, one of so much importance, so marked an influence having been exercised by the preceding editions of it on the state of ophthalmic medicine and surgery in this country, that we feel we should be neglecting our duty did we delay in giving a general notice of it at least.

As to the character of the work, it is not necessary for us to say much. The sale of two large editions in this country, the reprint of it at Boston in America, and the German translation of it published at Weimar, are sufficient proofs of its practical value. In the present edition, much new matter has been added: amongst other things, is an article on phlebitic ophthalmitis, and another on sympathetic ophthalmia, besides several new woodcuts; but, by enlarging the page, the work has been kept within its former bounds. "The whole," we are informed, "has again been carefully revised, and such alterations made as have been suggested by the author's continued experience, or by his perusal of the writings of others." "He trusts," the author further says, "that on every occasion he has endeavoured to treat his fellow-labourers in the same field with becoming deference and perfect fairness; never appropriating to himself the labours and improvements of others, but acknowledging openly what he has borrowed." Dr. Mackenzie's extremely full and accurate references are everywhere a proof of what is here expressed. Would we could say his fellow-labourers have always been as ingenuous towards him!

In regard to the anatomical part of the work by Mr. Wharton Jones, we are informed in the advertisement that, for this third edition, the author "has revised his Horizontal Section of the Eyeball, and Anatomical Introduction, comprehending in the latter the most recent discoveries in the anatomy of the eye." The anatomy of the appendages of the eye is not given. The sketch of the anatomy of the eyeball is brief but comprehensive; it and the figure of the horizontal section are mutually illustrative. For what is wanting in detail, full references are given to other works. In conclusion, Dr. Mackenzie's work may be truly characterized as the most complete treatise, theoretical and practical, on the diseases of the eye extant in any language.

